# Age- and AMH-based prediction of mature oocyte yield in oncofertility is well calibrated overall, but substantial underestimation is more frequent in hematologic malignancies

**DOI:** 10.3389/fonc.2026.1940599

**Published:** 2026-10-02

**Authors:** Claudia Massarotti, Sara Stigliani, Paola Scaruffi, Fausta Sozzi, Andrea Amaroli, Paola Anserini

**Affiliations:** 1 University of Genova, Dipartimento di Neuroscienze- Riabilitazione- Oftalmologia- Genetica e Scienze Materno-Infantili (DINOGMI Department), Genova, Italy; 2 AOM IRCCS Ospedale Policlinico San Martino, Clinica Ostetrica e Ginecologica, Genova, Italy; 3 AOM – IRCCS Ospedale Policlinico San Martino, SSD Physiopathology of Human Reproduction, Genova, Italy; 4 University of Genova, BIO-Photonics Overarching Research Laboratory (BIOPHOR), Department of Earth, Environmental and Life Sciences (DISTAV), Genova, Italy

**Keywords:** cancer and fertility, fertility preservation, hematologic cancers, oocytes cryopreservation, solid tumors

## Abstract

**Introduction:**

age and ovarian reserve markers are commonly used in clinical practice to predict ovarian response to controlled stimulation for assisted reproductive techniques in infertile patients. In oncofertility, some reports suggest the predictive performance of these markers may be lower in specific subgroups. We evaluated the performance of a prespecified age + log(AMH) model for predicting metaphase-II (MII) oocyte yield in women undergoing oocyte cryopreservation before gonadotoxic treatment.

**Methods:**

This retrospective single-center cohort study included consecutive oocyte cryopreservation cycles performed before gonadotoxic treatment between January 2015 and January 2025. MII yield was modelled using negative-binomial regression with age and log-transformed AMH as predictors. Regression coefficients were estimated in the current cohort. Model performance was assessed using calibration measures and patient-level bootstrap internal validation, with all cycles from the same woman kept together during resampling. Analyses were performed overall and by cancer type. Threshold-based underestimation analyses and the incremental value of antral follicle count (AFC) were also explored.

**Results:**

A total of 179 oocyte cryopreservation cycles performed in 167 women were included. The model showed good overall calibration using aggregated out-of-bootstrap predictions, with calibration-in-the-large of 0.00 (95% CI −0.08 to 0.09) and calibration slope of 0.98 (95% CI 0.80 to 1.15). Mean prediction error was −0.10 MII (95% CI −0.97 to 0.80), indicating minimal average directional bias. Mean absolute error was 4.12 MII (95% CI 3.53 to 4.76), and root mean squared error was 5.94 MII (95% CI 4.92 to 7.00). Substantial underestimation, defined as observed MII yield exceeding predicted yield by more than 4 MII, was more frequent in hematologic malignancies than in solid tumors (31.8% [14/44] vs 12.6% [17/135]; risk difference +19.2 percentage points, 95% CI + 4.4 to +34.1; Fisher’s exact p=0.006). This finding remained consistent in the one-cycle-per-woman sensitivity analysis. Adding AFC did not improve predictive performance in the complete-case analysis.

**Conclusion:**

age and AMH are useful tools for counseling regarding the expected mature oocyte yield, including in our subset of oncofertility patients. As we found substantial underestimation of mature oocyte yield to be more frequent in hematologic malignancies, the results should be interpreted cautiously in this subgroup.

## Introduction

Fertility preservation is now a routine part of cancer care in women of reproductive age (1). As referral pathways have improved and oocyte cryopreservation has become more widely integrated into clinical practice, counselling has also changed. The issue is no longer only whether fertility preservation can be offered in time, but also how to provide realistic, individualized expectations about the likely outcome of a cycle. In this setting, the number of mature oocytes cryopreserved is one of the most relevant endpoints, because it directly informs the expected reproductive value of treatment (2).

In routine reproductive medicine, age and anti-Müllerian hormone (AMH) are commonly used to estimate ovarian response and support counselling on expected oocyte yield (3, 4). They are simple, widely available, and easy to interpret in daily practice. In oncofertility, however, their performance may not be the same across all diagnoses (5). Women referred for fertility preservation are a heterogeneous population, and disease-related factors may influence ovarian reserve markers or stimulation outcomes even before the start of gonadotoxic treatment. This concern is particularly relevant in hematologic malignancies, where previous studies have suggested greater variability in ovarian reserve assessment and treatment response, with some studies reporting better results compared to those expected considering ovarian reserve markers and others no difference (6, 7).

It is therefore important to determine whether an age- and AMH-based model provides reliable estimates of mature oocyte yield in oncofertility, and whether its performance is consistent across oncologic subgroups. This is clinically relevant because a model may appear acceptable on average while still showing larger individual prediction errors, directional bias or clinically meaningful underestimation in specific patient groups. In such cases, predictions could lead to unrealistic expectations or, conversely, to overly pessimistic counselling about the potential benefit of fertility preservation.

We therefore evaluated the performance of a prespecified age + log(AMH) model for predicting the number of mature oocytes cryopreserved in women undergoing fertility preservation before gonadotoxic treatment. We assessed calibration, patient-level out-of-bootstrap prediction error, and substantial underestimation in the overall cohort and by cancer type. We also explored whether adding antral follicle count (AFC) improved predictive performance beyond age and AMH.

## Materials and methods

### Study design and population

This is a retrospective single-center cohort study conducted at a university hospital referral center for oncofertility. We included all consecutive oocyte cryopreservation cycles performed between January 2015 and January 2025 in women referred for fertility preservation before the start of any gonadotoxic treatment. Both solid and hematologic malignancies were included. Complete data on age, AMH and mature metaphase II oocytes (MII) yield were available for all included cycles. AFC was not required for inclusion in the primary analysis and was handled in a complete-case exploratory analysis.

The unit of analysis was the cycle. Women who underwent more than one consecutive oocyte cryopreservation cycle before treatment initiation were retained in the main analysis. Repeated cycles were addressed through patient-level bootstrap resampling and a one-cycle-per-woman sensitivity analysis.

Clinical and laboratory data were retrieved from routine medical records. These included age at the time of stimulation, cancer diagnosis, AMH, AFC, type and duration of controlled ovarian stimulation and the number of mature oocytes cryopreserved. Cancer diagnoses were grouped as solid tumors or hematologic malignancies.

### Ovarian stimulation and outcome definition

The controlled ovarian stimulation was carried out according to good clinical practice standards. The protocol used was the antagonist protocol for all cycles. The type and dosage of gonadotropins were chosen based on women’s age and ovarian reserve. GnRH antagonist was added when the leading follicle had a diameter of at least 12–13 mm and/or estradiol levels were >200 pg/mL. In women with hormone-sensitive breast cancer letrozole 5 mg/day was co-administered during the ovarian stimulation. Final oocyte maturation was triggered with GnRH agonist when leading follicles reached a diameter of 18–20 mm. Oocyte retrieval was performed transvaginally under ultrasound guidance, followed by assessment of oocyte maturity and cryopreservation according to local laboratory procedures. As this is a monocentric study, all cycles were carried out under the supervision of the same expert physician, guaranteeing uniformity in clinical choices.

AMH was measured using Elecsys AMH Plus automated one-step sandwich chemiluminescence immunoassay on a Cobas Pro e801 module (Roche Diagnostics).

AFC was measured using a Philips Affiniti 70 (Philips, Italy) with a 5–9 MHz transvaginal transducer, by counting all follicles measuring 2–9 mm in diameter.

The primary outcome was the number of mature oocytes cryopreserved in each cycle, defined as metaphase II (MII) oocytes.

### Statistical analysis

Continuous variables were reported as mean and standard deviation or median and interquartile range, as appropriate, and compared using Student’s t test or the Mann–Whitney U test. Categorical variables were reported as number and percentage and compared using the chi-square test or Fisher’s exact test, as appropriate.

The predictor set and functional form were prespecified before model fitting. Mature oocyte yield was modelled using age and log-transformed AMH as predictors in a negative-binomial regression model with log link, given the count nature of the outcome and the presence of overdispersion. AMH was transformed using the natural logarithm. No AMH value was equal to zero or below zero; therefore, no offset or imputation was required before log transformation. Age was entered as a continuous linear predictor on the model scale. No externally derived prediction equation with fixed intercept and coefficients was applied; therefore, the intercept and regression coefficients were estimated in the current cohort.

The model was intentionally limited to age and AMH because both variables are routinely available before treatment initiation and are commonly used in pre-cycle counselling. Variables related to stimulation management, such as gonadotropin dose, stimulation duration, trigger type, or protocol characteristics, were not included because they are partly determined after the initial clinical assessment. Accordingly, the model was intended to estimate expected MII yield under the clinical stimulation practices applied in this center, rather than a treatment-independent biological ovarian response.

Model performance was assessed in terms of calibration, directional bias, and individual-level prediction error. Calibration was evaluated using calibration-in-the-large (CITL), calibration slope, and calibration plots. Prediction error was calculated as observed minus predicted MII yield; therefore, positive values indicated underestimation by the model and negative values indicated overestimation. Mean prediction error was used to describe directional bias, whereas mean absolute error, median absolute error, and root mean squared error were used to quantify the magnitude of individual-level prediction error.

To obtain patient-level out-of-sample estimates of model performance, we used bootstrap internal validation with 2,000 bootstrap replicates. Resampling was performed at the patient level, so that all cycles from the same woman were kept together within each bootstrap sample. For each bootstrap replicate, the model was fitted in the bootstrap sample and predictions were generated for patients not included in that replicate, yielding out-of-bootstrap predictions. For each cycle, the final out-of-bootstrap predicted MII yield was obtained by averaging all out-of-bootstrap predictions available for that cycle. Performance measures were then calculated using these aggregated out-of-bootstrap predictions. Confidence intervals for prediction-error metrics were obtained by bootstrap resampling.

Women who underwent more than one consecutive oocyte cryopreservation cycle before treatment initiation were retained in the main analysis. The negative-binomial model did not include random effects or cluster-robust standard errors. To address the possible influence of repeated cycles, bootstrap resampling was performed at the patient level, and a sensitivity analysis restricted to one cycle per woman was also performed.

Subgroup analyses evaluated the performance of this same overall model within cycles from women with solid tumors and hematologic malignancies; the model was not refitted separately within each cancer group. Between-group differences in prediction error were assessed using the same bootstrap framework.

To complement average error metrics, we performed an exploratory threshold-based analysis of substantial underestimation. The primary pragmatic threshold was defined as observed MII yield exceeding predicted MII yield by more than 4 MII. This threshold was not intended to represent a validated clinical cutoff, but rather an error magnitude potentially relevant to counselling and less likely to reflect trivial variation of 1–2 oocytes. To assess robustness to threshold choice, we repeated the analysis using alternative thresholds of >3 and >5 MII. Between-group differences were reported using absolute risk difference, risk ratio, and Fisher’s exact test.

Finally, we explored whether adding AFC to the age + log(AMH) model improved predictive performance. AFC was evaluated in an exploratory complete-case analysis. Within the identical AFC complete-case cohort, the base age + log(AMH) model and the AFC-extended age + log(AMH) + AFC model were compared using the same patient-level out-of-bootstrap validation framework used for the primary analysis. In each bootstrap replicate, both models were fitted in the bootstrap sample and predictions were generated for patients not included in that replicate. For each cycle, aggregated out-of-bootstrap predictions were obtained separately for the base and AFC-extended models. Model performance was compared using mean prediction error, mean absolute error, median absolute error, root mean squared error, and the frequency of underestimation >4 MII.

Missing AFC values mainly reflected incomplete recording in routine medical charts during urgent baseline assessments performed in rapid fertility preservation pathways. As the analysis was retrospective, these values could not be reliably reconstructed. Cycles with and without AFC were compared descriptively.

All analyses were performed in R version 4.3.3 (R Foundation for Statistical Computing, Vienna, Austria). A two-sided p value <0.05 was considered statistically significant where applicable.

### Ethics

This study is part of a larger project on oncofertility, which was conducted in accordance with the Declaration of Helsinki and was approved by the local ethics committee (“Comitato Etico Regione Liguria, n. 428/2021”). Patients gave consent for the main study and for the use of their pseudonymized data for research. No reconsent was requested for this specific analysis.

## Results

### Study population

A total of 179 oocyte cryopreservation cycles performed in 167 women were included in the analysis. Of these, 155 women underwent one cycle and 12 underwent two consecutive cycles before the start of gonadotoxic treatment. Overall, 135 cycles (75.4%) were performed in women with solid tumors and 44 cycles (24.6%) in women with hematologic malignancies.

The mean age at stimulation was 32.58 years (SD 5.60). Median AMH was 1.88 ng/mL (Q1–Q3, 0.98–3.30), and median AFC was 12 (Q1–Q3, 7–18). Baseline characteristics of the overall cohort and by cancer group are reported in **Table 1**, together with stimulation data.

**Table 1:** Baseline characteristics of the study population.

Variable	Overall	Solid tumors	Hematologic malignancies	P value
Women, n	167	126	41	
Cycles, n	179	135	44	
Age, mean (SD), years	32.58 (5.60)	34.05 (4.50)	28.05 (6.22)	<0.001
AMH, median (Q1–Q3), ng/mL	1.88 (0.98–3.30)	1.83 (0.97–3.30)	2.14 (1.26–3.08)	0.425
AFC, median (Q1–Q3)	12 (7–18)	11 (6–17)	15 (11–20)	0.019
Random-start protocol, n/N (%)	88/161 (54.7%)	68/120 (56.7%)	20/41 (48.8%)	0.468
Total gonadotropin dose, mean (SD), IU	2971.64 (979.32)	3078.82 (939.11)	2657.74 (1037.59)	0.023
Stimulation length, mean (SD), days	11.78 (3.75)	11.76 (2.93)	11.83 (5.53)	0.937

Unless otherwise specified, characteristics are reported at the cycle level. Data are presented as mean (standard deviation), median (Q1–Q3), or n/N (%), as appropriate. P values compare solid tumors and hematologic malignancies and are descriptive only. AMH, anti-Müllerian hormone; AFC, antral follicle count; IU, international units.

Women with hematologic malignancies were younger than women with solid tumors (mean age 28.05 years [SD 6.22] vs 34.05 years [SD 4.50], p<0.001). AMH values were similar between groups (median 2.14 ng/mL [Q1–Q3, 1.26–3.08] vs 1.83 ng/mL [Q1–Q3, 0.97–3.30], p=0.425), whereas AFC was higher in hematologic malignancies (median 15 [Q1–Q3, 11–20] vs 11 [Q1–Q3, 6–17], p=0.019). Total gonadotropin dose was lower in hematologic malignancies than in solid tumors, while stimulation length and use of random-start protocols were similar between groups (**Table 1**).

### Overall model performance

Using aggregated patient-level out-of-bootstrap predictions, the prespecified age + log(AMH) model did not show major systematic miscalibration within the available data (see **Table 2**). Calibration-in-the-large was 0.00 (95% CI −0.08 to 0.09), indicating no relevant systematic over- or underprediction in the overall cohort. The calibration slope was 0.98 (95% CI 0.80 to 1.15), supporting acceptable agreement between predicted and observed MII yield across the prediction range.Mean observed MII yield was 10.16 (95% CI 9.06 to 11.23), and mean out-of-bootstrap predicted MII yield was 10.25 (95% CI 9.49 to 11.04). Mean prediction error, calculated as observed minus predicted MII yield, was −0.10 MII (95% CI −0.97 to 0.80), indicating minimal average directional bias.

**Table 2:** Predictive performance of the age + log(AMH) model using aggregated out-of-bootstrap predictions.

Metric	Overall	Solid tumors	Hematologic malignancies
Cycles, n	179	135	44
Women, n	167	126	41
Mean observed MII	10.16 (9.06 to 11.23)	9.46 (8.33 to 10.69)	12.30 (10.04 to 14.75)
Mean OOB predicted MII	10.25 (9.49 to 11.04)	9.87 (9.02 to 10.77)	11.44 (10.17 to 12.85)
Mean prediction error, MII	−0.10 (−0.97 to 0.80)	−0.41 (−1.41 to 0.54)	0.86 (−0.96 to 2.75)
Mean absolute error, MII	4.12 (3.53 to 4.76)	3.85 (3.16 to 4.55)	4.97 (3.80 to 6.31)
Median absolute error, MII	2.87 (2.15 to 3.35)	2.67 (2.06 to 3.19)	4.27 (2.00 to 5.53)
Root mean squared error, MII	5.94 (4.92 to 7.00)	5.73 (4.40 to 6.93)	6.53 (4.92 to 8.20)
Underestimation >4 MII	17.3% (31/179)	12.6% (17/135)	31.8% (14/44)

Values are based on aggregated patient-level out-of-bootstrap predictions. Prediction error was calculated as observed minus predicted MII yield; positive values indicate underestimation. Confidence intervals were obtained by bootstrap resampling. Substantial underestimation was defined as observed MII yield exceeding predicted MII yield by more than 4 MII. AMH, anti-Müllerian hormone; MII, metaphase II oocytes; OOB, out-of-bootstrap.

Individual-level prediction error was moderate. Mean absolute error was 4.12 MII (95% CI 3.53 to 4.76), median absolute error was 2.87 MII (95% CI 2.15 to 3.35), and root mean squared error was 5.94 MII (95% CI 4.92 to 7.00). The calibration plot showed acceptable agreement between predicted and observed MII yield in the overall cohort, although estimates at the extremes of the prediction range should be interpreted cautiously because fewer observations were available in these regions (**Figure 1A**).

**Figure 1:**
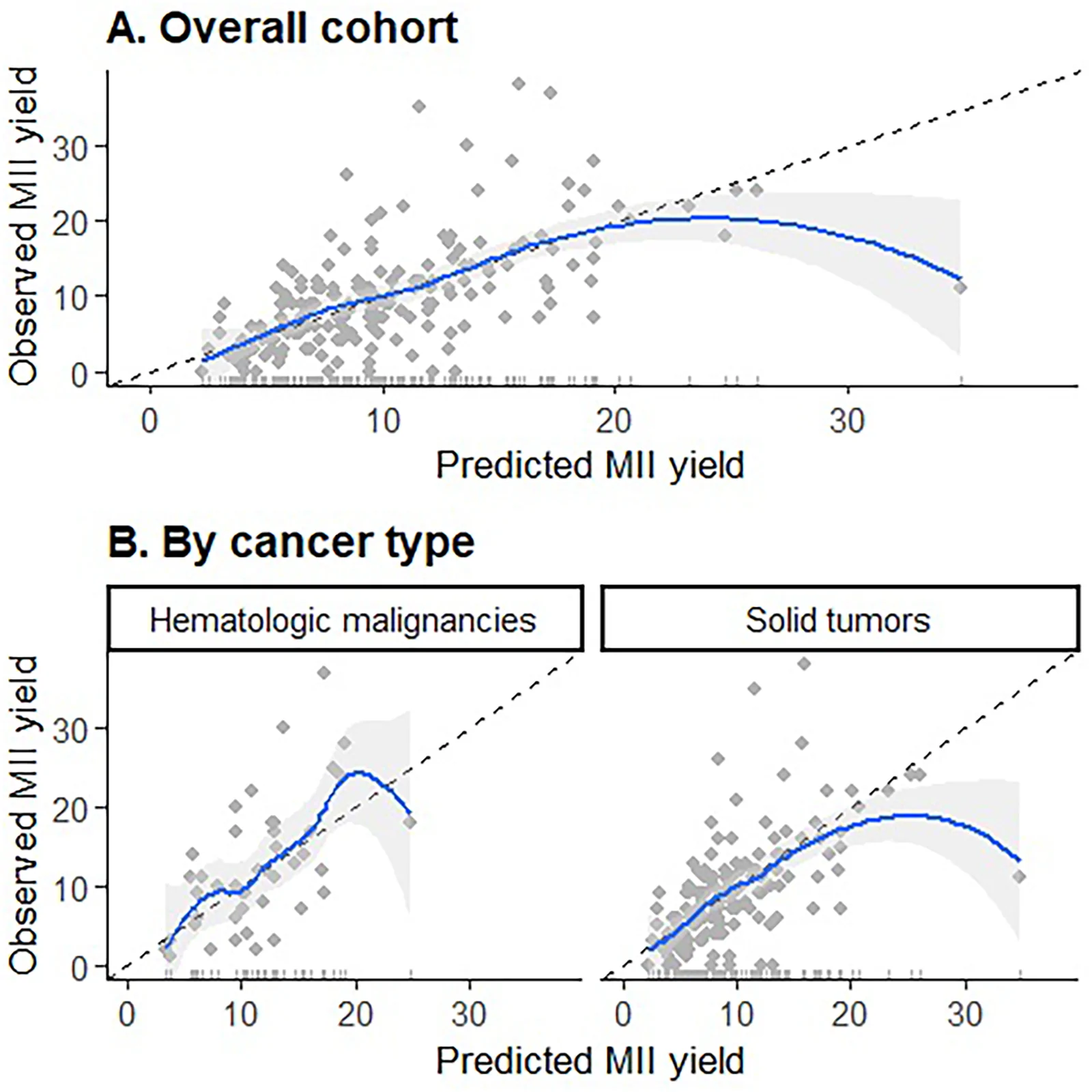
Calibration plots of the age + log(AMH) model for prediction of mature oocyte yield. Panel **(A)** shows calibration in the overall cohort. Panel **(B)** shows calibration stratified by cancer type. Predicted values were based on aggregated patient-level out-of-bootstrap predictions. The dashed diagonal line represents perfect agreement between predicted and observed MII yield. Points represent individual cycles. The blue smoothed curves show the observed relationship between predicted and observed values, and shaded bands represent 95% confidence bands around the smoothed calibration estimates. Rug plots indicate the distribution of predicted values. Calibration at the extremes of the prediction distribution should be interpreted cautiously because fewer observations were available in these regions, particularly within the hematologic malignancy subgroup.

Model coefficients are reported in **Supplementary Table S1**, and out-of-bootstrap calibration estimates are reported in **Supplementary Table S2**.

To assess whether repeated cycles influenced the results, we repeated the analysis after restricting the dataset to one cycle per woman (n=167 women; 167 cycles). In this sensitivity analysis, results remained similar (**Supplementary Table S3**). Mean prediction error was −0.07 MII, mean absolute error was 4.04 MII, median absolute error was 2.78 MII, and root mean squared error was 5.82 MII. Calibration-in-the-large was 0.00 (95% CI −0.08 to 0.08), and the calibration slope was 1.00 (95% CI 0.83 to 1.17).

### Model performance by cancer type

When model performance was evaluated by cancer type using aggregated out-of-bootstrap predictions, mean observed MII yield was 9.46 (95% CI 8.33 to 10.69) in solid tumors and 12.30 (95% CI 10.04 to 14.75) in hematologic malignancies. Mean out-of-bootstrap predicted MII yield was 9.87 (95% CI 9.02 to 10.77) and 11.44 (95% CI 10.17 to 12.85), respectively.

Mean prediction error was −0.41 MII (95% CI −1.41 to 0.54) in solid tumors and 0.86 MII (95% CI −0.96 to 2.75) in hematologic malignancies. The estimated between-group difference in mean prediction error, hematologic malignancies minus solid tumors, was +1.27 MII (95% CI −0.86 to 3.35). Thus, although the direction of average error suggested mild underestimation in hematologic malignancies and mild overestimation in solid tumors, the confidence interval for the between-group difference included zero.

Individual-level prediction error was numerically higher in hematologic malignancies. Mean absolute error was 4.97 MII (95% CI 3.80 to 6.31) in hematologic malignancies and 3.85 MII (95% CI 3.16 to 4.55) in solid tumors. Root mean squared error was 6.53 MII (95% CI 4.92 to 8.20) and 5.73 MII (95% CI 4.40 to 6.93), respectively. The estimated between-group differences were +1.12 MII for mean absolute error (95% CI −0.37 to 2.57) and +0.80 MII for root mean squared error (95% CI −1.30 to 2.84).

In the exploratory threshold-based analysis, substantial underestimation was more frequent in hematologic malignancies than in solid tumors. Using the primary pragmatic threshold of underestimation >4 MII, this occurred in 31.8% of hematologic malignancy cycles and 12.6% of solid tumor cycles (14/44 vs 17/135), corresponding to a risk difference of +19.2 percentage points (95% CI + 4.4 to +34.1) and a risk ratio of 2.53 (95% CI 1.36 to 4.70; Fisher’s exact p=0.006). This finding was consistent in the one-cycle-per-woman sensitivity analysis, where underestimation >4 MII occurred in 34.1% of hematologic malignancy cycles and 13.5% of solid tumor cycles (risk difference +20.7 percentage points, 95% CI + 5.0 to +36.3; Fisher’s exact p=0.005).

Robustness analyses using alternative thresholds showed a consistent direction of effect. Underestimation >3 MII occurred in 31.8% of hematologic malignancy cycles and 18.5% of solid tumor cycles (Fisher’s exact p=0.091), whereas underestimation >5 MII occurred in 27.3% and 10.4%, respectively (Fisher’s exact p=0.012). Results of the threshold robustness analyses are reported in **Supplementary Table S4**.

Calibration plots stratified by cancer type showed broadly acceptable agreement between predicted and observed MII yield in both groups, although estimates in the hematologic malignancy subgroup and at the extremes of the prediction range should be interpreted cautiously because of the smaller number of observations in these regions (**Figure 1B**).

### Incremental value of AFC

AFC was available for 154 cycles from 143 women, corresponding to 86.0% of the cohort, and was evaluated in an exploratory complete-case analysis. Missing AFC values mainly reflected incomplete recording during urgent baseline visits and could not be retrospectively recovered. Cycles with missing AFC did not show clear differences from cycles with available AFC in age, AMH, MII yield, cancer type distribution, or random-start protocol use. In the complete-case AFC-extended model, AFC was not independently associated with MII yield after accounting for age and AMH. The AFC coefficient was 0.005 (95% CI −0.009 to 0.018), corresponding to an incidence rate ratio of 1.005 (95% CI 0.991 to 1.018; p=0.495).

In the patient-level out-of-bootstrap complete-case comparison, adding AFC did not improve predictive performance. Mean absolute error was 4.30 MII with the base model and 4.39 MII with the AFC-extended model, while median absolute error was 3.00 and 3.13 MII, and root mean squared error was 6.25 and 6.34 MII, respectively. Underestimation >4 MII occurred in 28/154 cycles (18.2%) with the base model and 29/154 cycles (18.8%) with the AFC-extended model. In the paired comparison, underestimation >4 MII was reclassified in only three cycles: one cycle was classified as underestimation >4 MII by the base model only, and two cycles by the AFC-extended model only (exact paired p=1.000).

Detailed AFC complete-case analyses, including the out-of-bootstrap comparison of base and AFC-extended models and the comparison of cycles with and without AFC, are reported in **Supplementary Table S5**.

## Discussion

At the first oncofertility consultation, clinicians are required to discuss complex and uncertain scenarios with patients who have recently received a cancer diagnosis and often need to make fertility-related decisions within a short timeframe. In this setting, age and ovarian reserve markers are used not only to estimate the potential gonadotoxic impact of treatment, but also to support counselling about the expected yield of a fertility preservation cycle (8).

In routine reproductive medicine, AMH is a well-established marker of quantitative ovarian response (3), whereas age remains the strongest marker of oocyte competence and reproductive potential (4, 9). Together, these variables provide a clinically intuitive framework for estimating expected oocyte yield. However, in oncofertility, this framework may be less straightforward (10). Patients referred before gonadotoxic treatment represent a heterogeneous population, and disease-related factors, timing of referral, systemic symptoms, inflammation, or differences in baseline clinical characteristics may affect ovarian reserve markers or stimulation response (6).

In this study, we evaluated a prespecified age + log(AMH) model structure for predicting mature oocyte yield in women undergoing fertility preservation before any gonadotoxic treatment. The model was not an externally fixed equation; rather, the predictor set and functional form were prespecified, and the regression coefficients were estimated in the current cohort. Using patient-level out-of-bootstrap internal validation, the model showed good overall calibration and minimal average directional bias. These findings support the use of age and AMH as useful baseline counselling tools for estimating expected mature oocyte yield in oncofertility.

However, the subgroup analysis showed a clinically relevant nuance. Although average prediction error did not differ conclusively between cancer types, substantial underestimation was more frequent in hematologic malignancies. Using the primary pragmatic threshold of underestimation >4 MII, nearly one third of cycles in women with hematologic malignancies were underestimated, compared with approximately one in eight cycles in women with solid tumors. This pattern remained consistent in the one-cycle-per-woman sensitivity analysis and in threshold robustness analyses using alternative cutoffs.

This finding does not imply that the age + log(AMH) model should be abandoned in hematologic malignancies. Rather, it suggests that predictions in this subgroup should be communicated with particular caution. The model can provide an expected mature oocyte yield, but clinicians should avoid presenting this estimate as a precise individual prediction. This is especially relevant in fertility preservation, where the difference between obtaining, for example, 4–5 versus 9–10 mature oocytes may influence how patients perceive the potential value of undergoing stimulation and oocyte cryopreservation.

The higher frequency of substantial underestimation in hematologic malignancies may have several explanations. Women in this subgroup were younger and had higher AFC values than women with solid tumors, indicating that the two groups occupied different regions of the predictor space. Therefore, the observed pattern may reflect cancer type itself, differences in case mix and predictor distribution, disease-related or inflammatory factors, urgency and timing of referral or a combination of these mechanisms. The present study was not designed to isolate a causal effect of cancer type on prediction error, and the relatively small hematologic subgroup limited the possibility of robust interaction analyses.

Our findings therefore support non-deterministic counselling when age and AMH are used to discuss expected mature oocyte yield. In particular, low or intermediate ovarian reserve markers should not be interpreted as precise individual forecasts of poor oocyte yield. This is especially relevant in hematologic malignancies, where a subset of patients obtained substantially more mature oocytes than predicted by age and AMH alone. The clinical implication is not that AMH should be disregarded, but that its interpretation should remain probabilistic and contextual, particularly in urgent oncofertility pathways.

We also explored whether adding AFC improved prediction beyond age and AMH. AFC was not independently associated with MII yield after accounting for age and AMH and the AFC-extended model did not improve AIC, mean absolute error, root mean squared error, or the frequency of underestimation >4 MII. This does not mean that AFC is biologically irrelevant. Rather, AFC and AMH may provide partly overlapping information on quantitative ovarian reserve. From a pragmatic perspective, the lack of clear incremental value of AFC is relevant because AFC may be operator-dependent.

The main strength of this study is that it evaluated a prespecified and clinically intuitive predictor set and functional form, rather than using data-driven variable selection or adding predictors based on their apparent performance in the same dataset. We also assessed model performance using patient-level out-of-bootstrap prediction, allowing calibration, directional bias, individual-level prediction error, and clinically interpretable underestimation to be considered as distinct aspects of predictive performance. This is particularly important in oncofertility counselling, where a model may be well calibrated on average but still underestimate yield in a clinically meaningful subset of patients.

This study also has limitations. First, it is a single-center retrospective analysis. Predicted MII yield should therefore be interpreted as the expected outcome under this center’s stimulation practices, rather than as a treatment-independent biological ovarian response. External validation is needed before the model or its calibration can be generalized to other centers or stimulation strategies. Second, although patient-level bootstrap resampling and a one-cycle-per-woman sensitivity analysis were used to address repeated cycles, a small number of women contributed more than one cycle in the main analysis. Third, the hematologic subgroup was relatively small, and subgroup findings should therefore be interpreted cautiously. Fourth, AFC was not available for all cycles and its incremental value was assessed in an exploratory complete-case analysis. Finally, the outcome was mature oocyte yield rather than subsequent reproductive outcomes, pregnancy, or live birth. The clinical interpretation should therefore be restricted to counselling about expected oocyte yield and uncertainty around that estimate.

Future studies should externally validate age- and AMH-based prediction of mature oocyte yield in larger multicenter oncofertility cohorts, with particular attention to hematologic malignancies. Such studies should determine whether recalibration, cancer-specific adjustment or additional predictors can reduce substantial underestimation in this subgroup. Ultimately, the goal should be to translate ovarian reserve markers into counselling tools that are statistically reliable, clinically interpretable and usable in urgent fertility preservation pathways.

## Data Availability

The raw data supporting the conclusions of this article will be made available by the authors, without undue reservation.
