# Supplementary material for: Age- and AMH-based prediction of mature oocyte yield in oncofertility is well calibrated overall, but substantial underestimation is more frequent in hematologic malignancies
Source: Front Oncol. 2026 Oct 2;16:1940599. doi: 10.3389/fonc.2026.1940599 (PMC13671969; doi:10.3389/fonc.2026.1940599)
Supplement: Supplementary file 1 [file Table1.docx]

**Supplementary Tables**

**Supplementary Table S1. Main negative-binomial model**

| **Predictor** | **Estimate** | **Standard error** | **z value** | **p value** | **IRR** | **IRR 95% CI** |
| --- | --- | --- | --- | --- | --- | --- |
| Intercept | 2.617 | 0.270 | 9.678 | <0.001 | 13.694 | 8.061 to 23.265 |
| Age | -0.021 | 0.008 | -2.715 | 0.007 | 0.979 | 0.964 to 0.994 |
| log(AMH) | 0.517 | 0.054 | 9.522 | <0.001 | 1.676 | 1.507 to 1.865 |

**Note:** The model was fitted using negative-binomial regression with log link. AMH was transformed using the natural logarithm. IRR, incidence rate ratio.

**Model characteristics**

| **Characteristic** | **Value** |
| --- | --- |
| Cycles, n | 179 |
| Women, n | 167 |
| Theta | 4.773 |
| Theta SE | 0.820 |
| AIC | 1083.294 |
| Log-likelihood | -537.647 |
| 2 x log-likelihood | -1075.294 |
| Null deviance | 334.998 |
| Residual deviance | 206.440 |
| Null degrees of freedom | 178 |
| Residual degrees of freedom | 176 |

**Supplementary Table S2. Out-of-bootstrap calibration**

| **Metric** | **Estimate** | **SE** | **95% CI** |
| --- | --- | --- | --- |
| CITL | 0.000 | 0.043 | -0.084 to 0.085 |
| Calibration slope | 0.975 | 0.090 | 0.799 to 1.151 |

**Note:** Calibration was assessed using aggregated patient-level out-of-bootstrap predictions. CITL was estimated using a negative-binomial model with the log of the predicted MII yield as offset. Calibration slope was estimated by regressing observed MII yield on the log of predicted MII yield.

**Supplementary Table S3. One-cycle-per-woman sensitivity analysis**

**a.Model coefficients**

| **Predictor** | **Estimate** | **Standard error** | **z value** | **p value** | **IRR** | **IRR 95% CI** |
| --- | --- | --- | --- | --- | --- | --- |
| Intercept | 2.685 | 0.262 | 10.250 | <0.001 | 14.662 | 8.774 to 24.502 |
| Age | -0.022 | 0.008 | -2.877 | 0.004 | 0.978 | 0.964 to 0.993 |
| log(AMH) | 0.496 | 0.052 | 9.445 | <0.001 | 1.642 | 1.481 to 1.820 |

**b.Model characteristics**

| **Characteristic** | **Value** |
| --- | --- |
| Cycles, n | 167 |
| Women, n | 167 |
| Solid tumors, n | 126 |
| Hematologic malignancies, n | 41 |
| Theta | 5.451 |
| Theta SE | 0.988 |
| AIC | 1012.560 |
| Log-likelihood | -502.280 |
| 2 x log-likelihood | -1004.560 |
| Null deviance | 318.060 |
| Residual deviance | 191.420 |
| Null degrees of freedom | 166 |
| Residual degrees of freedom | 164 |

**c.Predictive performance**

| **Metric** | **Estimate** | **95% CI** |
| --- | --- | --- |
| Mean observed MII | 10.57 |  |
| Mean predicted MII | 10.64 |  |
| Mean prediction error, MII | -0.07 |  |
| Mean absolute error, MII | 4.04 |  |
| Median absolute error, MII | 2.78 |  |
| Root mean squared error, MII | 5.82 |  |
| CITL | 0.000 | -0.082 to 0.082 |
| Calibration slope | 1.000 | 0.826 to 1.174 |

**d.Threshold sensitivity analysis**

| **Threshold** | **Solid tumors** | **Hematologic malignancies** | **Risk difference, percentage points (95% CI)** | **Risk ratio (95% CI)** | **Fisher p value** |
| --- | --- | --- | --- | --- | --- |
| >3 MII | 25/126 (19.8%) | 14/41 (34.1%) | 14.3 (-1.8 to 30.4) | 1.72 (0.99 to 2.99) | 0.088 |
| >4 MII | 17/126 (13.5%) | 14/41 (34.1%) | 20.7 (5.0 to 36.3) | 2.53 (1.37 to 4.67) | 0.005 |
| >5 MII | 14/126 (11.1%) | 12/41 (29.3%) | 18.2 (3.2 to 33.1) | 2.63 (1.33 to 5.23) | 0.011 |

**Note:** In the one-cycle-per-woman sensitivity analysis, one cycle per woman was retained and the age + log(AMH) model was refitted in this restricted dataset. Prediction-error and threshold-based metrics were calculated using predictions from this refitted sensitivity model, rather than by restricting the primary-analysis predictions to first cycles. This analysis was intended to assess the possible influence of repeated cycles. AMH, anti-Müllerian hormone; MII, metaphase II oocytes.

**Supplementary Table S4. Threshold robustness analysis using out-of-bootstrap predictions**

| **Threshold** | **Solid tumors** | **Hematologic malignancies** | **Risk difference, percentage points (95% CI)** | **Risk ratio (95% CI)** | **Fisher p value** |
| --- | --- | --- | --- | --- | --- |
| >3 MII | 25/135 (18.5%) | 14/44 (31.8%) | 13.3 (-1.9 to 28.5) | 1.72 (0.98 to 3.00) | 0.091 |
| >4 MII | 17/135 (12.6%) | 14/44 (31.8%) | 19.2 (4.4 to 34.1) | 2.53 (1.36 to 4.70) | 0.006 |
| >5 MII | 14/135 (10.4%) | 12/44 (27.3%) | 16.9 (2.8 to 31.0) | 2.63 (1.32 to 5.25) | 0.012 |

**Note:** Threshold analyses were based on aggregated patient-level out-of-bootstrap predictions. Prediction error was calculated as observed minus predicted MII; positive values indicate underestimation. Thresholds represent exploratory cutoffs for substantial underestimation and were evaluated as sensitivity analyses around the primary pragmatic threshold of >4 MII.

**Supplementary Table S5. AFC complete-case analysis**

a. **AFC complete-case dataset**

| **Characteristic** | **Value** |
| --- | --- |
| Cycles with AFC available, n | 154 |
| Women with AFC available, n | 143 |
| Percentage of total cycles | 86.0% |
| Solid tumors, n | 117 |
| Hematologic malignancies, n | 37 |

b. **AFC coefficient in the extended model**

| **Parameter** | **Estimate** | **Standard error** | **95% CI** | **IRR** | **IRR 95% CI** | **p value** |
| --- | --- | --- | --- | --- | --- | --- |
| AFC | 0.005 | 0.007 | -0.009 to 0.018 | 1.005 | 0.991 to 1.018 | 0.495 |

c. **Model performance: base vs AFC-extended model**

| **Metric** | **Base model: age + log(AMH)** | **Extended model: age + log(AMH) + AFC** | **Difference, extended minus base** |
| --- | --- | --- | --- |
| Mean observed MII | 9.95 (8.83 to 11.10) | 9.95 (8.83 to 11.10) | 0.00 (0.00 to 0.00) |
| Mean OOB predicted MII | 10.08 (9.23 to 10.93) | 10.11 (9.23 to 11.00) | 0.04 (−0.05 to 0.13) |
| Mean prediction error, MII | −0.13 (−1.13 to 0.85) | −0.16 (−1.17 to 0.82) | −0.04 (−0.13 to 0.05) |
| Mean absolute error, MII | 4.30 (3.60 to 5.07) | 4.39 (3.69 to 5.17) | 0.09 (0.01 to 0.18) |
| Median absolute error, MII | 3.00 (2.45 to 3.54) | 3.13 (2.50 to 3.66) | 0.13 (−0.13 to 0.32) |
| Root mean squared error, MII | 6.25 (5.08 to 7.44) | 6.34 (5.19 to 7.55) | 0.09 (0.00 to 0.21) |
| Underestimation >4 MII, n | 28 | 29 | 1 |
| Underestimation >4 MII, % | 18.2% | 18.8% | 0.6 pp (−1.3 to 2.7) |

d. **Paired comparison for underestimation >4 MII**

| **Characteristic** | **Value** |
| --- | --- |
| Neither model classified as underestimation >4 MII | 124 |
| Base model only | 1 |
| Extended model only | 2 |
| Both models | 27 |
| Difference in underestimation >4 MII, percentage points | 0.6 |
| Exact paired/binomial p value | 1.000 |

e. **AFC available vs AFC missing**

| **AFC status** | **Cycles, n** | **Women, n** | **Cancer type distribution** | **Age, mean (SD)** | **AMH, median (Q1-Q3)** | **MII, mean (SD)** | **MII, median (Q1-Q3)** |
| --- | --- | --- | --- | --- | --- | --- | --- |
| AFC available | 154 | 143 | 117 solid tumors (76.0%); 37 hematologic malignancies (24.0%) | 32.75 (5.50) | 1.86 (0.98-3.22) | 9.95 (7.49) | 9.00 (5.00-13.00) |
| AFC missing | 25 | 24 | 18 solid tumors (72.0%); 7 hematologic malignancies (28.0%) | 31.52 (6.18) | 2.13 (1.10-3.47) | 11.44 (6.28) | 11.00 (8.00-14.00) |
| **Comparison** | | | | **p value** | | | |
| Age | | | | 0.357 | | | |
| AMH | | | | 0.668 | | | |
| MII | | | | 0.141 | | | |
| Cancer type | | | | 0.626 | | | |
| Random start | | | | 0.757 | | | |

**Note:** AFC was unavailable in 25 cycles. Missing AFC values mainly reflected incomplete recording during urgent baseline assessments and could not be retrospectively recovered. AFC was evaluated in an exploratory complete-case analysis.

The AFC coefficient was estimated from the AFC-extended model fitted in the complete-case sample. Prediction-performance measures were calculated using aggregated patient-level out-of-bootstrap predictions obtained separately for the base and AFC-extended models within the identical AFC complete-case cohort. Prediction error was calculated as observed minus predicted MII yield; positive values indicate underestimation. Confidence intervals were obtained by patient-level bootstrap resampling.

Comparisons between AFC-available and AFC-missing cycles are descriptive and exploratory.

AFC, antral follicle count; AMH, anti-Müllerian hormone; MII, metaphase II oocytes; OOB, out-of-bootstrap.
